# CORESIDENCE: National and subnational data on household size and composition around the world, 1964–2021

**DOI:** 10.1038/s41597-024-02964-3

**Published:** 2024-01-31

**Authors:** Juan Galeano, Albert Esteve, Anna Turu, Joan García-Roman, Federica Becca, Huifen Fang, Maria Pohl, Rita Trias-Prats

**Affiliations:** 1 Universitat Autònoma de Barcelona/Centre d’Estudis Demogràfics, Barcelona, Spain; 2 Centre d’Estudis Demogràfics, Barcelona, Spain

**Keywords:** Society, Sociology

## Abstract

The CORESIDENCE Database (CoDB) represents a significant advancement in the field of family studies, addressing existing data gaps and facilitating comprehensive analysis of households’ composition and living arrangements at the national and subnational levels. This article introduces the CoDB, developed for the ERC project Intergenerational Coresidence in Global Perspective: Dimensions of Change. The database draws on global-scale individual microdata from four main repositories and national household surveys, encompassing over 150 million individual records representing more than 98% of the world’s population. The CoDB provides datasets at the national, subnational, and subnational-harmonized levels, covering 156 countries, 3950 regions, and 1511 harmonized regions for the period 1964–2021. It includes 146 indicators on household composition and family arrangements, allowing researchers to explore intergenerational co-residence patterns, gender dynamics within households, and longitudinal trends in living arrangements. The CoDB fills an important gap in comparative household studies, enabling researchers to undertake ground breaking research at both macro and micro levels, ultimately fostering a deeper understanding of the complex dynamics of family structures and living arrangements.

## Background & Summary

Households represent the most fundamental unit of human organization. They play a crucial role in child-rearing, elderly care, resource allocation, and shaping gender roles^1^. While the composition of households is primarily based on familial bonds, practices vary significantly across societies. Factors such as demographics^2^, economics, and social norms^3^ influence variations in household size and composition. Consequently, households have significant implications for social reproduction, urbanization, housing demands, and consumption. Despite their significance, the availability of household level data at the global scale is underdeveloped and could be substantially expanded thanks to the increasing availability of household level microdata. To bridge this information gap, the Coresidence database (CoDB) provides access to 146 harmonized indicators on household size and composition for 156 countries, 3950 subnational areas, and 58 data points in time. Compared to the United Nations database on Household Size and Composition (https://www.un.org/development/desa/pd/data/household-size-and-composition), CoDB complements, updates, and introduces new features. Firstly, CoDB exclusively includes data from countries where microdata is accessible to researchers. While this slightly limits the number of countries compared to the United Nations database, it significantly expands analytical possibilities, as this microdata can subsequently be use to explore living arrangements at the household levels. Secondly, by leveraging microdata, CoDB broadens the number of indicators on household size and composition. A total of 146 indicators have been calculated covering the following domains: indicators related to size and age composition of households, indicators derived from the relation of family members to the person defined as the head of the household, indicators related to household’s typology and indicators related to household headship. Thirdly, CoDB offers detailed subnational insights by preserving the original and changing regions within each sample but also providing indicators across a standardized set of harmonized regions. Finally, the open-source code in R utilized in this project is publicly accessible, allowing users to observe how the microdata has been processed and indicators have been built. This ensures replicability and empowers users to create new indicators.

CoDB has been developed within the project “Intergenerational Coresidence in Global Perspective: Dimensions of Change (CORESIDENCE)”, funded by the European Research Council. The available indicators in CoDB have been calculated from individual microdata samples from four large data repositories of international microdata, supplemented by national household surveys. All included samples allow grouping individuals into households and examining the relationships established among their members. Additionally, they provide basic sociodemographic information about household members, including age, sex, and marital status. With all the samples combined, the original microdata database contains more than 150 million individual records, representing more than 98% of the world’s population and spanning from the 1960’s to the present. The 146 indicators contained in CoDB represent different aggregations of the original microdata, both by country and subnational areas. Within each country, subnational areas have been harmonized to facilitate the study of change over time. As a final output, CoDB consists of three datasets: The National dataset contains 156 countries, the Subnational dataset contains 3950 subnational areas, and the Subnational harmonized dataset contains 1511 subnational areas for the period 1964 to 2021, and it provides 146 indicators on household composition and family arrangements across the world.

## Methods

### Overview

**Fig. 1:**
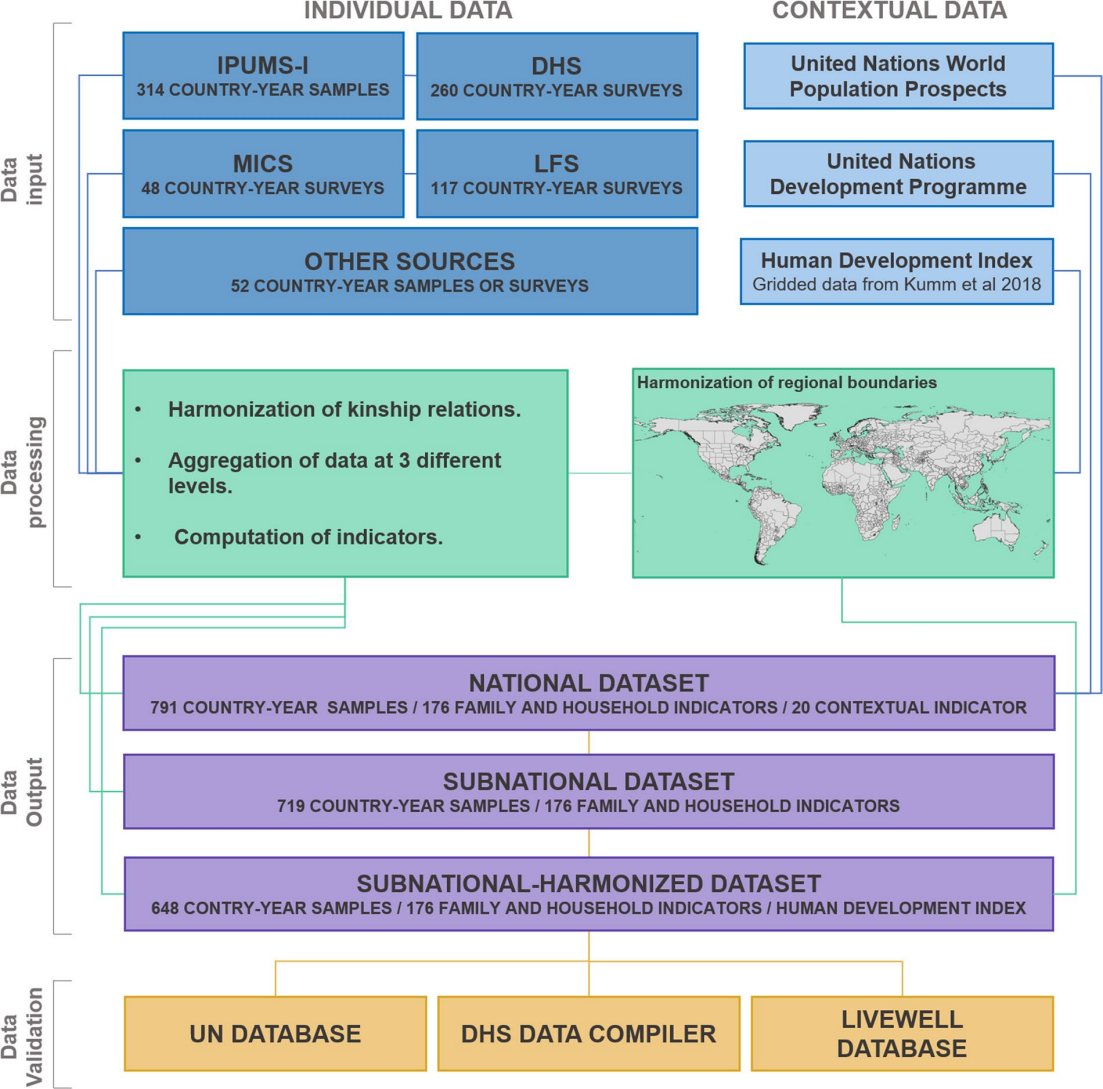
Flowchart representing the different stages to build the CoDB.

Figure 1 provides a schematic overview of the entire process of creating CoDB, starting with data acquisition, and followed by data processing, harmonization, indicator’s construction, output datasets, and external validation. CoDB draws on four main repositories of global-scale individual microdata: The International Integrated Public Use Microdata Series (IPUMS-I), the Demographic Health Surveys (DHS), the Multiple Indicator Cluster Surveys (MICS), and the European Union Labor Force Survey (EU-LFS). Additionally, CoDB includes country-specific surveys and censuses not available in any of the previous repositories, such as the EU Statistics on Income and Living Conditions (EU-SILC) surveys, the Income and Labour Dynamics in Australia (HILDA) surveys, the Household Income and Expenditure Survey (HIES) for South Korea and the China Family Panel Studies (CFPS). Contextual indicators come directly from various UN datasets, specifically the United Nations World Population Prospects^4^ (UNWPP), the United Nations Development Programme^5^ (UNDP) and from gridded data of the Human Development Index from Kumm *et al*.^6^.

All data cleaning, processing, harmonization and aggregation were performed in R^7^. For the Subnational harmonized dataset, the use of QGIS^8^ was additionally required. All the coded used in the construction of CoDB is available in the GitHub repository of this project (see section Code availability).

The output data of CoDB includes three datasets: National, Subnational, and Subnational harmonized.

**Fig. 2:**
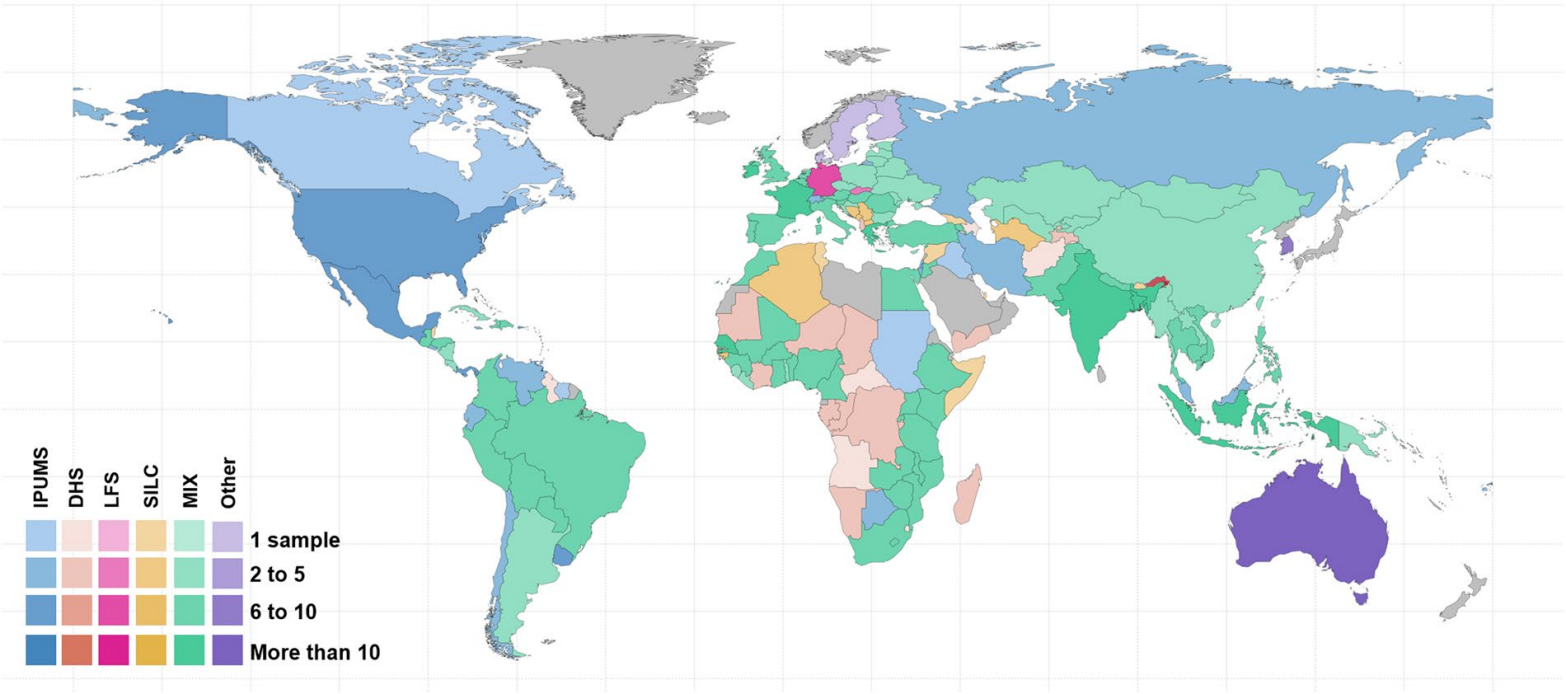
Country coverage by number of samples available of the CoDB.

The National dataset includes 791 country-year samples from 155 countries (Fig. 2). Figure 2 provides an overview of the number of countries included in the database and the data available for each of them. For each sample, Fig. 2 informs about the source of reference and about what type of subnational data is available per sample. The National dataset contains 146 indicators on household size and composition worldwide for over 60 years. The selected indicators provide information on the size and composition of households. Regarding composition, details are provided on the age, relationship to the reference person, type of household (e.g. unipersonal, nuclear, extended), and sex of the reference person in the households (see section 1.4). These are standard measures in household research using similar data sources^9^. This dataset incorporates an additional set of 20 contextual indicators obtained from the UNWPP and the UNDP. These additional indicators provide information on population size, life expectancy by sex, fertility rates, and the human development index for each country in a given year.

The Subnational dataset includes 719 country-year samples covering 149 countries and 3,950 unique regions. The 146 indicators were calculated based on the major administrative unit in which households were enumerated in each of the primary data sources. Out of the original 791 samples, 72 were not included in this dataset due to the absence of territorial disaggregation information (see Fig. 2).

**Fig. 3:**
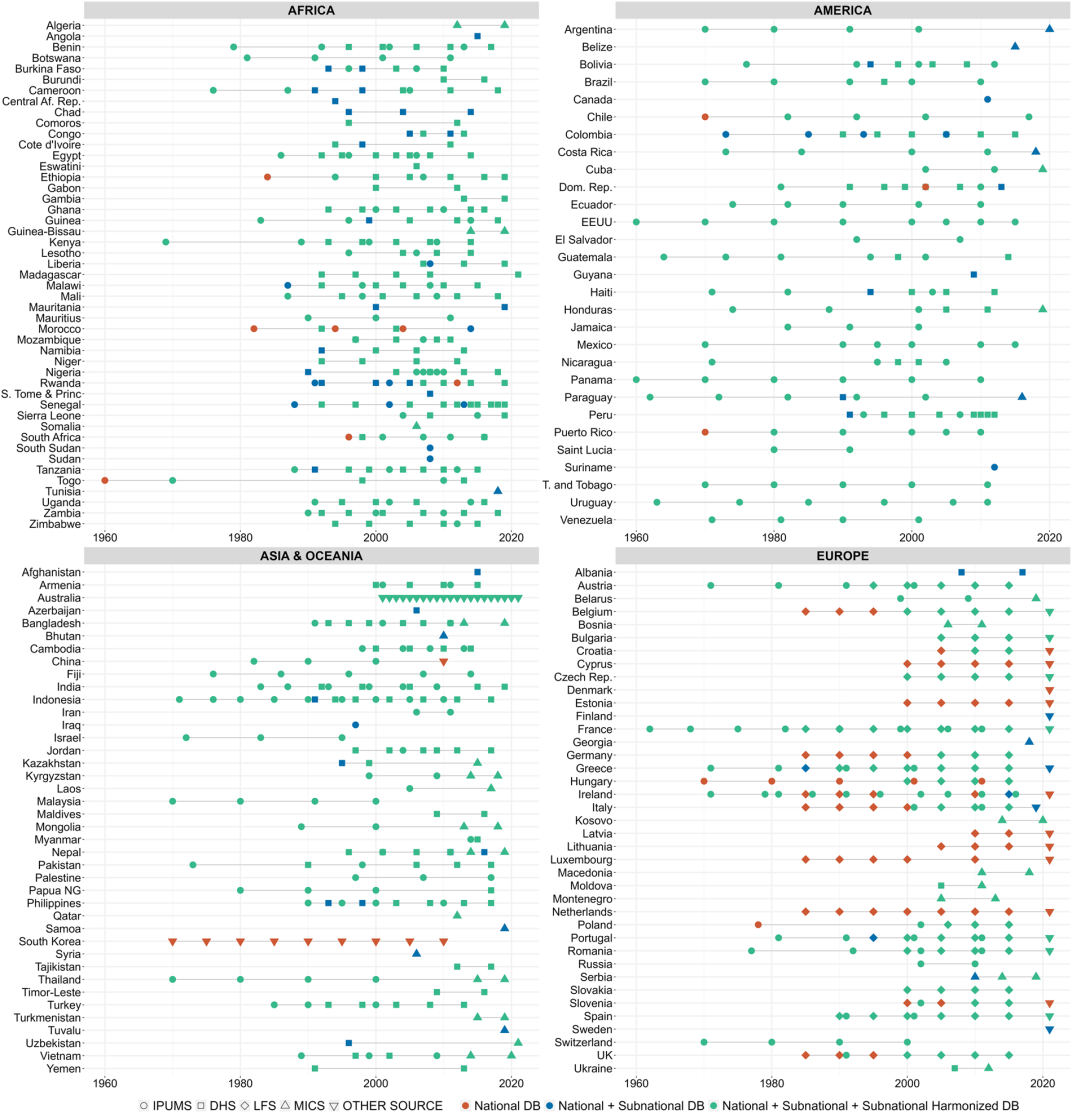
Availability of samples by country, year, and source in the CoDB.

Last, the Subnational Harmonized dataset consists of 648 country-year samples from 138 countries and 1,511 unique regions. To ensure consistency and minimize repetition, only countries for which we had more than one sample and regions could be harmonized over time were included. As a result, the Subnational Harmonized dataset covers 82% of the original samples. Figure 3 shows the regional breakdown available in the Subnational Harmonized dataset (Fig. 3 in green). The regions marked in green are present in this database. For countries with only on sample (e.g. Canada), it is necessary to retrieve the data from the Subnational dataset. Regarding the indicators, the same 146 are available for all these regions. The harmonization of geographic boundaries is explained in sub-section Harmonization of regional subnational boundaries. Regarding contextual data, indicators such as life expectancy or fertility are not available at this scale. However, data from the Human Development Index, extracted through from Kumm *et al*. (2008)^3^, has been included (see section Contextual indicators).

In addition to the three datasets, the CoDB also provides a spatial file with the boundaries of the subnational harmonized regions either as *sf* object or a multi-polygon geopackage (see section Data Records). For the production of the spatial file, we relied on the already harmonized geographies provided by the IPUMS international, the DHS Spatial Repository^10^, the work done by the LiveWell project^11^ for harmonizing DHS boundaries and the Database of Global Administrative Areas (GADM)^12^.

To ensure the accuracy and reliability of the CoDB, we validated our database by comparing the results of a selected set of indicators from the three datasets with corresponding data from reputable sources such as the UN database on Household Size and Composition^13^, the DHS STAT compiler^14^ and the LiveWell project (see section 3 on Technical Data Validation).

### Data sources

The CoDB is a comprehensive source of information on household structure and family composition at national and subnational levels. The database draws on four major repositories of individual microdata on a global scale, along with country-specific surveys and censuses from countries not included in those repositories. Additionally, we employed external data sources to provide a set of contextual (demographic and socioeconomic) indicators.

The first source of individual microdata for the CoDB is the International Integrated Public Use Microdata Series (IPUMS-I)^15^, consisting of 314 census samples from 94 countries (https://international.ipums.org). The IPUMS International project is a global initiative that aims to collect, preserve, harmonize, and distribute census microdata from countries worldwide. In all cases, except for countries with fewer households in a specific year, a sample of 20,000 households from the original microdata was randomly selected to build the CoDB indicators. This was done to minimize data storage and speed processing, but users can rebuild these indicators with larger samples using the same source. In the validation process (see section 3), we show that our estimates are consistent with those of the United Nations based on indicators that are available in both UN and the CoDB sources.

The second source of individual microdata for the CoDB is the Demographic Health Surveys (DHS)^16^ (https://dhsprogram.com/data/), which have been collecting demographic and health information for low- and middle-income countries since 1986. A total of 260 samples from 75 countries were retrieved. DHS surveys rely on a two-stage cluster sampling design that ensures the representativeness of the data at the national and subnational level.

To expand the coverage of the CoDB beyond the countries and years included in the two previous repositories, 49 additional samples from 33 countries were included from the Multiple Indicator Cluster Surveys (MICS) program^17^ (https://mics.unicef.org/surveys), which collects data related to key indicators of health, education, child protection, and water and sanitation. MICS surveys are designed to collect data at both national and subnational levels. The data is publicly available and has been widely utilized for studying family structures and change in a variety of countries.

Microdata from 117 samples of the European Labour Force Survey (EU-LFS)^18^ were used to complement the information available on European countries from IPUMS (https://ec.europa.eu/eurostat/web/microdata/european-union-labour-force-survey). The EU-LFS is a large household sample survey on the labour force participation of the 15-year and older population, also collecting information on all members of the household surveyed, as well as the kinship relations among them. As LFS collects data on a quarterly basis, samples included in the CoDB correspond to the yearly samples to ensure consistency with the specific time frame for which the data was downloaded.

The CoDB includes information from country-specific surveys and censuses for countries and/or years not present in the previous repositories. This includes: 22 samples of the EU Statistics on Income and Living Conditions (EU-SILC) survey^19^ for the year 2021 (https://ec.europa.eu/eurostat/web/microdata/eu-silc), 21 samples from the Household, Income and Labour Dynamics in Australia (HILDA)^20^ survey between 2001 and 2021 (https://melbourneinstitute.unimelb.edu.au/hilda), 9 samples from the South Korean Census (http://kosis.kr/eng/) covering the period 1970–2010 and 2 sample from the China Family Panel Studies (CFPS)^21^ for the years 2010 and 2018 (https://www.isss.pku.edu.cn/cfps/en/).

Last, for the set of contextual socio-demographic indicators provided in the National dataset of the CoDB we used data from the United Nations World Population Prospects (UNWPP) (https://population.un.org/wpp/) and the United Nations Development Programme (UNDP) (https://hdr.undp.org/data-center). The UNWPP provides information on global population trends, projections, and demographic indicators, whereas the UNDP focuses on promoting human development globally. To get subnational estimates of the Human Development Index for the Subnational Harmonized dataset, we utilized the HDI gridded dataset developed by Kummu *et al*.^3^ (https://datadryad.org/stash/dataset/doi:10.5061/dryad.dk1j0).

The CoDB has been designed with a forward-looking perspective, poised to accommodate the ongoing growth of its constituent data repositories. As the aforementioned data sources continue to release new samples, the CoDB is primed to seamlessly integrate these additions, ensuring its comprehensiveness over time.

## Harmonization Processes

### Harmonization of household interrelationship variables

In the construction of the CoDB, a crucial step was the harmonization of relationships among household members from diverse data sources. Most of these relationships involve a certain degree of kinship, but the amount of detail varies widely. We followed the IPUMS-I harmonization coding scheme to harmonize intrahousehold relationships for the other sources.

The IPUMS-I samples include a harmonized variable called “relate” which captures 75 distinct types of relationships (or their absence) with respect to the reference person of the household, often named the household head. Not all types of relationships are present in every sample. The detailed classification of types is grouped into six categories: Head, Spouse/Partner, Child, Other relative, Other non-relative, and Other relative or non-relative.

In the case of DHS and MICS surveys, before establishing equivalences with the IPUMS-I categories, an additional step was necessary to harmonize the data internally, as the same kinship category was recorded in slightly different ways across different surveys. For instance, variations like “brother-in-law or sister-in-law”, “brother-in-law/sister-in-law”, and “brother-in-law/sister-in-law” were observed. Through the internal harmonization process, these variations were consolidated into 24 distinct categories for DHSs and 39 categories for MICSs. These categories were then aligned with the corresponding ones from the IPUMS-I samples.

The EU Labour Force Surveys (LFS) only capture 6 types of relations, but crucially for the purpose of this project the type: ‘Ascendant relative of reference person (or of his/her spouse or cohabiting partner)’ is included. The EU statistics on income and living conditions (EU-SILC) offer a broader perspective, encompassing 19 different types of relations to the head. In the case of South Korea, the census samples provided by the National Office of Statistics provide a wider range of recorded relations to the head, varying between 13 and 38 depending on the specific year.

In the case of the Australian data, the absence of a designated head or reference person made the procedure more complex. However, leveraging the available information on the total income of household members, we employed a specific criterion to define the head of the household. The person with the highest total income was identified as the head, ensuring consistency between the surveys provided by the National Statistical Institute of Australia. In the rare instances where two members had exactly the same income, the older person was designated as the head. Additionally, we had to re-code all the relations within the household as they were originally recorded from the perspective of the individual (ego) to all other members of the household, ensuring a consistent and standardized representation of kinship relations.

The Chinese Family Panel Survey (CFPS) also provides the relations between household members as a matrix of “all versus all” type. The source code for the re-coding of relations can be accessed and downloaded from the CORESIDENCE project’s GitHub repository (see section **Code Availability**). In total, 17 types of relations to the head were defined and aligned with IPUMS-I.

### Harmonization of regional subnational boundaries

One of the key and original features of the CoDB with respect to other databases is the provision of subnational data in the Subnational and the Subnational Harmonized datasets. For this latter one, geographical boundaries were harmonized to facilitate the study of change over time.

For harmonizing the subnational regions we relied on four major sources of spatial data information: the spatially harmonized first-level geography from IPUMS International (https://international.ipums.org/international/gis_harmonized_1st.shtml), the work done by the LiveWell project^8^ for the harmonization of DHS boundaries, the DHS Spatial Repository (http://spatialdata.dhsprogram.com/home/), and the Database of Global Administrative Areas, GADM (https://gadm.org/)

The harmonization process involved multiple steps. First, we selected countries with at least two data samples. Second, we identified the smallest common spatial denominator to allow for comparisons over time. Third, we categorized the selected countries based on whether all the data samples originated from the same data source or not. When all samples originated from the same source, we encountered two distinct scenarios. Firstly, if the samples were obtained from IPUMS, which already had a pre-existing harmonized subnational division and identification system, no further harmonization was needed. Secondly, when the data comes from sources other than IPUMS, it has been necessary to harmonize administrative boundaries in some countries. When all samples from a country come from the DHS, we assigned an IPUMS-like ID to each of the harmonized regions (6 digits where the 3 first digits are the ISO numeric code of the country), following the process developed by the LiveWell project. The same process was applied when all samples were obtained from the LFS or SILC. For the 21 samples from Australian HILDA data, the subnational regions were already harmonized and we only assigned a new ID to each region.

**Fig. 4:**
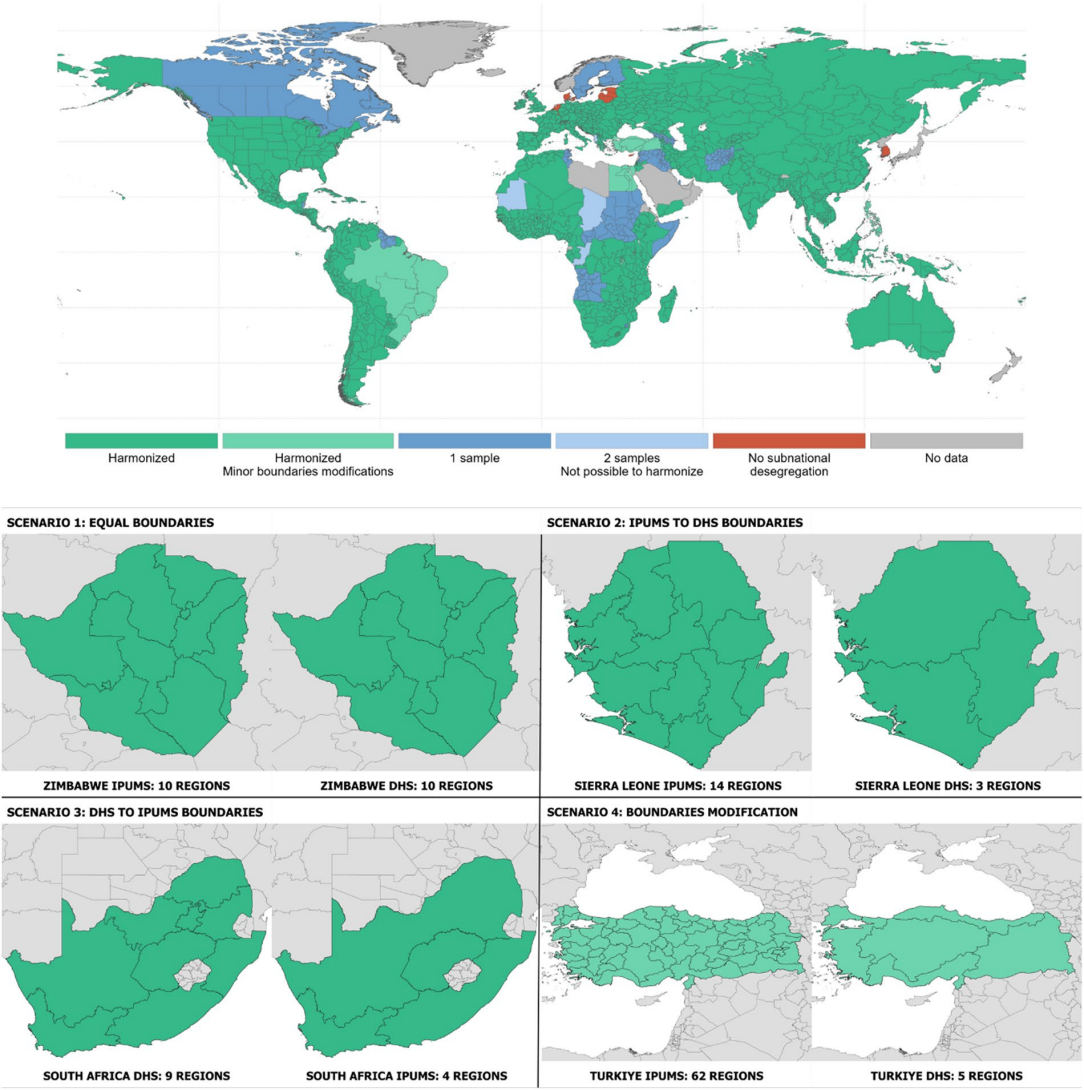
Harmonized Subnational coverage of the CoDB.

When dealing with samples from different data repositories for a given country, the harmonization process became more complex. Where there was a perfect match between sources, such as Zimbabwe, the harmonization process was straightforward (Fig. 4, scenario 1). In this country, both the IPUMS-I and DHS samples used the same regional breakdown of the country. In these instances, we used the GEOLEVEL1 IDs from IPUMS to harmonize the DHS data. In other cases, for instance that of South Africa or Sierra Leone, the harmonization process involved the aggregation of regions (Fig. 4, scenario 2 and 3). When aggregating data from DHS to IPUMS, we retained the region IDs provided by IPUMS. Conversely, when aggregating data from IPUMS to DHS, we created new IDs for both sources, as it was the case for countries with samples from the LFS and SILC repositories. The last scenario we encountered involved making slight modifications to regional boundaries between sources (Fig. 4, scenario 4). This was the case for samples from Turkey, Philippines, Egypt, and Brazil, and the affected regions are listed in the harmonization table provided within the CoDB.

The R code for re-coding the individual data of each sample to the harmonized regions can be found, consulted and downloaded from the GITHUB repository of the CORESIDENCE project (see section Code availability).

### Construction of indicators on household composition and living arrangements

From the original microdata presented in section 1.2., we have calculated 146 indicators on household composition and family arrangements globally over a span of 60 years. To generate these indicators, we aggregated the individual data to the national, subnational, and harmonized subnational levels across our three datasets. The data was weighted using the individual weights provided by each sample.

The indicators provided in the CoDB can be grouped into four categories: (i) indicators related to size and age composition of households, (ii) indicators derived from the relation of family members to the person defined as the head of the household, (iii) indicators related to household’s typology and (iv) indicators related to household headship. Before computing the indicators included in the CoDB, the population was weighted by the relevant survey weight (household or individual weight), ensuring representativeness with respect to the underlying population. This comprehensive set of indicators offers a rich resource for studying household composition and living arrangements across different contexts and time periods.

(i). *Indicators related to size and age composition of households*: The first set of indicators (HS01 to HS11) focuses on the relative distribution of households by size (ranging from 1 to 10 persons and 11 or more persons). To further explore the composition of households, indicators HS12 to HS14 provide information on the proportion of households with 2–3, 4–5, or 6 or more persons as computed in the UN database on Household Size and Composition; thus, enabling external validation of our own computations (see section on Technical Validation). Indicators HS15 and HS16 compute the proportion of households with at least one person aged 0–4 or 65 or more years old respectively. In the CoDB, this is presented as an average (HS17) at the national, subnational, or subnational harmonized level. Indicators HS18 to HS21 provide additional insights into the average number of persons in households, categorized by age groups: 0–4 years, below 18 years, above 18 years, and 65 years or older. These indicators shed light on the age distribution within households. Moreover, indicators HS22 to HS30 provide information on the average number of persons in households within 10-year age intervals. This allows for a more detailed understanding of the age composition of households.
(ii). *Indicators derived for the relation of family members to the person defined as the head or of the household*: These indicators offer insights into the structure and dynamics of family relationships. The first group of indicators (HR01 to HR06) provides information about the average number of heads, spouses, children, other relatives, and non-relatives in the household. These indicators help us understand the composition of the household in terms of these specific family relationships. Moreover, this information is further disaggregated based on the size of the household, specifically households with 2 to 5 people. Indicators HR07 to HR30 present the average number of heads, spouses, children, other relatives, and non-relatives in households of this size range. This allows for a more detailed analysis of the relationship dynamics within different household configurations. By examining these indicators, we can improve our understanding of the social structure and interdependencies among family members within households of various sizes. This information contributes to a deeper understanding of family dynamics and relationships within different contexts.
(iii). *Indicators related to household’s typology*: Indicators related to household typology in the CoDB offer valuable insights into the diverse forms and compositions of households across different contexts. To ensure comparability and overcome variations in the types of kinship relations recorded in the different data sources, we computed indicators based on two distinct typologies.

The first typology, developed by the CORESIDENCE team, consists of eight categories:

1. Unipersonal households.
2. Nuclear households: consisting of a head, a spouse, and their children, or a head and their children.
3. Nuclear households with additional relatives.
4. Nuclear households with non-relatives.
5. Nuclear households with both relatives and non-relatives.
6. Other relative households.
7. Other non-relative households.
8. Other households with a combination of relatives and non-relatives.

The second typology, based on the work of John Bongaarts^6^ for developing countries in the 1990s, comprises five categories:

1. Unipersonal households.
2. Nuclear households: consisting of a head, a spouse, and their children, or a head and their children.
3. Stem family additions: including parents or grandchildren of the head.
4. Other family households: encompassing other relatives of the head.
5. Other non-family households: comprising individuals not related to the head.

Using these two sets of typologies, the indicators (HT01 to HT31) provide information on the proportion and average size of each household type. These indicators shed light on the prevalence and characteristics of various household types, contributing to a deeper understanding of household structures and arrangements across different populations and time periods within the CoDB.

(iv). *Indicators related to household headship*: Indicators related to household headship in the CoDB capture important dimensions covered in the previous sets of indicators, such as proportions of *n* persons households, average sizes, and typologies. However, they specifically consider the gender dimension in relation to the household head (HH01 to HH56). These indicators provide key information on the roles and dynamics of gender within households. They shed light on the distribution of male-headed and female-headed households, offering a deeper understanding of how gender influences household structures and arrangements. By examining proportions, average sizes, and typologies of male-headed and female-headed households, these indicators contribute to a comprehensive analysis of household composition and dynamics, while considering gender dimension.

### Contextual indicators

In addition to the household level indicators, the National and Subnational harmonized datasets included in the CoDB provide contextual indicators. Within the National dataset, for each country-year sample included in the CoDB, we provide population counts, total fertility rates (TFR), and life expectancy by sex. In addition to demographic indicators, CoDB includes socio-economic measures, such as the Human Development Index (HDI) and its components. The HDI is a composite index that assesses the overall development and well-being of a country, considering factors such as life expectancy, education, and income. The components of HDI included in CoDB are: expected years of schooling, mean years of schooling, Gross Domestic Income (GDI), and Gross National Income (GNI) per capita. The socio-economic indicators are also divided by sex.

In the case of the Subnational Harmonized (SH) dataset, we utilized the HDI gridded dataset developed by Kummu *et al*.^3^ to provide the Human Development Index (HDI) at the subnational level for all the harmonized samples between 1990 and 2015 included in the CoDB. This allowed us to capture the variations in development within countries at a more detailed geographical level.

To calculate the average HDI values for each Subnational Harmonized region in our dataset, we proceeded as follows. First, we transformed the gridded HDI data for each year into a spatial points layer using the *“raster pixels to points”* function from the processing toolbox of QGIS. Next, we clipped the spatial boundaries of our SH dataset with the points shapefile by joining their attributes based on location. Finally, we summarized the joined data by the harmonized ID and year and computed the mean HDI values using R. This process allowed us to provide the HDI at the subnational level for 72.8% of the region-year entries of the SH dataset.

By including total fertility rates, life expectancy, and socio-economic indicators like the HDI, the CoDB empowers researchers and policymakers to explore the demographic and socio-economic landscapes of different countries and time periods in relation to changes in family arrangements and households’ composition. These indicators facilitate a deeper understanding of population dynamics, thereby supporting evidence-based decision-making and policy formulation.

## Data Records

The CoDB is hosted in Zenodo^22^, an open-access digital repository that allows researchers, scientists, and scholars from various disciplines to share and preserve their research outputs. Zenodo is operated by CERN (European Organization for Nuclear Research) and supported by various organizations, including the European Commission’s OpenAIRE project. The CoDB is hosted at the permanent 10.5281/zenodo.8142652^22^. The repository is composed of the following elements: a RData file named CORESIDENDE_DB containing the CoDB in the form of a List. In R, a List object is a versatile data structure that can contain a collection of different data types, including vectors, matrices, data frames, other lists, spatial objects or even functions. It allows to store and organize heterogeneous data elements within a single object. The CORESIDENDE_DB R-list object is composed of six elements:

1. NATIONAL: a data frame with the household composition and living arrangements indicators at the national level.
2. SUBNATIONAL: a data frame with the household composition and living arrangements indicators at the subnational level computed over the original subnational division provided in each sample and data source.
3. SUBNATIONAL_HARMONIZED: a data frame with the household composition and living arrangements indicators computed over the harmonized subnational regions.
4. SUBNATIONAL_BOUNDARIES_CORESIDENCE: a spatial data frame (a sf object) with the boundary’s delimitation of the subnational harmonized regions created for this project.
5. CODEBOOK: a data frame with the complete list of indicators, their code names and description.
6. HARMONIZATION_TABLE: a data frame with the full list of individual country-year samples employed in this project and their state of inclusion in the 3 datasets composing the CoDB.

Elements 1, 2, 3, 5 and 6 of the R-list are also provided as *csv* files under the same names. Element 4, the harmonized boundaries, is at disposal as *gpkg* (Geopackage) file.

## Technical Validation

To ensure the accuracy and reliability of the CoDB, we employ a two-stage validation process. In the first stage, we validate our National dataset by comparing some of our indicators to those from the DHS STAT compiler^11^ and the UN database on Household Size and Composition.

The DHS STAT compiler, developed by the DHS Program, is a user-friendly interface that facilitates the exploration and visualization of indicators derived from DHS survey data at the national and subnational levels. Complementing this, the United Nations (UN) database on Household Size and Composition serves as a comprehensive repository that gathers data from diverse sources to offer insights into the worldwide size and composition of households at the national level. By harmonizing and standardizing the measurement and classification of household characteristics, it enables comparisons and analysis across countries.

**Fig. 5:**
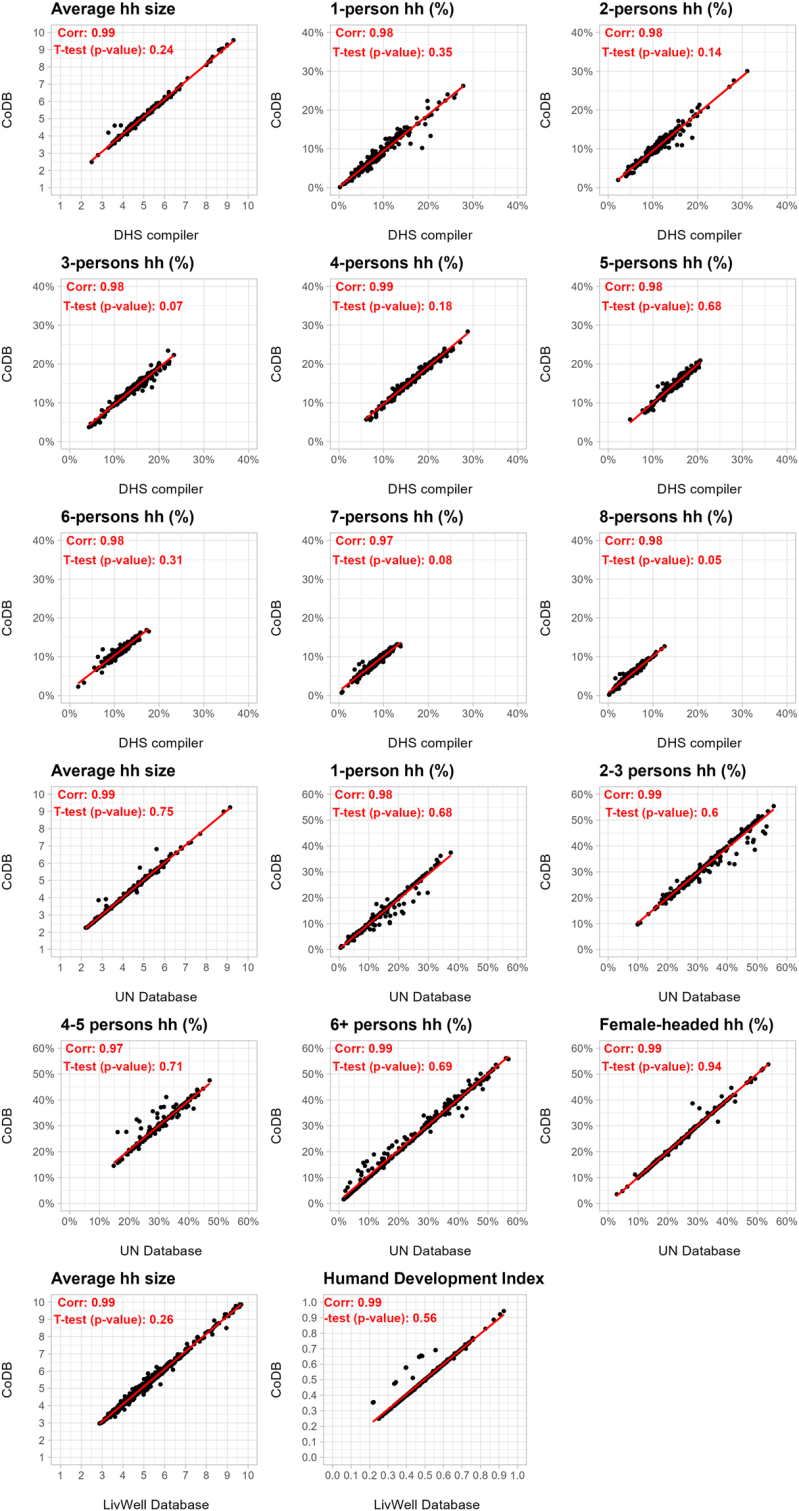
External validation of the CoDB.

Among the indicators provided by the STAT compiler at the national level, there is a specific set of nine indicators providing information on the average number of people per household and the relative distribution of them by size, which allow us to compare 255 surveys from 74 countries. Using the UN database, we compared 269 samples from IPUMS and 14 surveys from MICS, encompassing data from 91 countries, over a set of six indicators connected with the same dimensions plus the share of female-headed households. Leveraging these tools, we assess the consistency and alignment of our National dataset indicators with these two reputable sources, ensuring the reliability and validity of our data. Overall, the correlation between the country-level indicator of the CoDB and the ones from the STAT compiler and the UN database is highly linear, suggesting a good fit of our computations (Fig. 5). Additionally, we computed an equal variance T-test for each of the selected indicators. The p-values, greater than the common significance level of 0.05, suggest that the observed difference in means is likely due to random variation, primarily associated with the data cleaning and processing steps. This indicates that the disparities between the compared databases are more likely a result of data handling rather than genuine differences in means.

In the second stage, we validate the Subnational Harmonized dataset using data from the LiveWell project and the subnational human development database^23^. These additional sources of data enable us to cross-reference and corroborate the harmonized indicators at the subnational level. To validate the Subnational Harmonized dataset, we conducted the same analysis as for the National Database using three directly comparable indicators sourced from the LiveWell database. This validation process encompassed 1485 region-year entries, accounting for approximately 20.4% of our dataset. This validation process is crucial to ensure the robustness, accuracy, and overall quality of our subnational harmonized dataset, as well as to support its usefulness for demographic analysis and/or to inform policy decision-making Fig. 5.

## Data Availability

The processing steps to build the three datasets composing the CoDB were carried out in R, utilizing the libraries tidyerse^24^, haven^25^, labelled^26^, and tibble^27^. All the code is available on the GitHub repository of this project: https://github.com/JuanGaleano/CORESIDENCE.
